# Pericentric inversion (Inv) 9 variant—reproductive risk factor or benign finding?

**DOI:** 10.1007/s10815-019-01601-y

**Published:** 2019-11-16

**Authors:** Katrina Merrion, Melissa Maisenbacher

**Affiliations:** Natera, Inc., San Carlos, CA USA

**Keywords:** Structural rearrangement, Pericentric inversion of chromosome 9, In vitro fertilization (IVF), Preimplantation genetic testing for structural rearrangements (PGT-SR), Aneuploidy

## Abstract

**Purpose:**

To report the unbalanced chromosome rearrangement rate and overall aneuploidy rate in day 5/6 embryos from a series of patients who underwent in vitro fertilization (IVF) with preimplantation genetic testing for structural rearrangements (PGT-SR) for the pericentric inversion 9 variant, inv(9)(p11q13) or inv(9)(p12q13), with concurrent 24 chromosome preimplantation genetic testing for aneuploidy (PGT-A).

**Methods:**

This was a retrospective cohort analysis. IVF cycles and embryo biopsies were performed by referring clinics. Fifty-two trophectoderm biopsy samples from seven couples were sent to a single lab for PGT-SR for an inversion 9 variant with concurrent 24 chromosome PGT-A using single-nucleotide polymorphism (SNP) microarrays with bioinformatics.

**Results:**

The unbalanced rearrangement rate for this embryo cohort was 0/52 (0.0%); mean maternal age per embryo was 33.3 years (range 21–39 years). The overall euploid rate was 61.5% and aneuploidy rate was 38.5%.

**Conclusions:**

Chromosome 9 pericentric inversions did not result in unbalanced structural rearrangements in day 5/6 embryo samples, supporting that this population variant is not associated with increased reproductive risks.

## Introduction

Pericentric inversion 9 is a common chromosome variant with an incidence of approximately 1.6% in the general population [1]. Despite the relatively high incidence of this finding, there is debate in the literature over its clinical significance resulting in confusion about how to counsel patients regarding the medical management of this variant. By definition, a pericentric inversion involves a rearrangement of chromosome material that includes the centromere, and during meiosis can result in gametes with an unbalanced distribution of chromosome material. The chromosome imbalance results from formation of recombinant chromosomes following a crossover event between the inversion and the normal homolog of the chromosome. The resulting gametes either have a duplication involving the distal segment of the short arm (p-arm) of the chromosome and a deletion of the distal segment of the long arm (q-arm) of the chromosome, or vice versa [2]. These unbalanced chromosomes may result in non-viable embryos, early miscarriages, or livebirths with multiple anomalies. The risk for abnormal chromosome segregation is dependent on the chromosome involved, the location and size of the rearranged chromosome material, and the sex of the carrier. Some authors have proposed that pericentric inversions can also result in an interchromosomal effect, as described in Anton et al. [3], which influences the segregation and aneuploidy rates of other chromosomes. In general, the risk for a pericentric inversion to result in a child with an unbalanced chromosomal rearrangement ranges from 1–10% [2].

The pericentric inversion 9 variant is reportedly an exception to chromosome rearrangements that cause non-disjunction and by some authors has been noted to carry no increased reproductive risk [2, 4, 5], while other literature continues to suggest a clinical consequence associated with this inversion [6–12]. The pericentric inversion 9 variant involves the heterochromatic region of chromosome 9 and can occur with varying breakpoints. The high frequency of variants involving this region has been purported to relate to repetitive DNA sequences and homology of the 9p12 and 9q13 regions [13]. Inversion (9)(p11q13) has been widely referenced in the literature but the most common breakpoints have been reported to involve (9)(p12p13) [13].

Conflicting reports on the clinical significance of this inversion fuel the debate on the necessity of PGT-SR for couples with a carrier of the inversion. Some publications indicate the pericentric inversion 9 variant can be associated with unbalanced rearrangements [14], infertility [6–8], recurrent spontaneous abortions [9], and abnormal phenotype [10–12]. While others regard it as a benign population variant [2, 4, 5], supported by studies reporting no abnormalities in infants born with this inversion [15].

These contradictory reports have supported certain patients’ decisions to pursue preimplantation genetic testing for structural rearrangements (PGT-SR) for the inversion 9 variant during in vitro fertilization (IVF) cycles. To our knowledge, there has only been one prior publication to report on aneuploidy rates in embryos of inversion 9 carriers using preimplantation genetic testing for aneuploidy (PGT-A) [14]. Our study differs as we utilized both PGT-SR to assess for unbalanced rearrangements of the inversion and concurrent 24 chromosome PGT-A to assess for overall aneuploidy rates in preimplantation embryo samples from carriers of the inversion 9 variant. This analysis of the embryo chromosome results was performed to determine whether the inversion 9 variant carries a reproductive risk that should be considered a referral indication for PGT-SR with an IVF cycle.

## Methods

This was a retrospective cohort study. All PGT-SR cases received between April 2013 and May 2018 for the indication of a parental inversion 9 variant, as confirmed by standard karyotype analysis, were included in the study. Before accepting embryo samples for testing, the parents' karyotype reports, which indicated either inv(9)(p11q13) or inv(9)(p12q13), were reviewed to determine that there was adequate single-nucleotide polymorphism (SNP) coverage on the microarray to detect the potential unbalanced chromosome products that could result from the common inversion. Trophectoderm (TE) embryo biopsies were performed according to each IVF clinic’s standard procedures on day 5/6 following fertilization. TE biopsy samples and parental samples were shipped to a single reference laboratory for genotyping using Illumina Cyto12 SNP microarrays with Parental Support^TM^ [16]. This methodology incorporates parental SNP genotype information to predict the possible SNP genotypes for an embryo. For each chromosome, the algorithm compares the observed SNP data to each of the predicted allele distributions for each copy number hypothesis and identifies the one with the maximum likelihood. Embryo samples were compared with parent samples across multiple SNP loci to determine parental origin of each chromosome, rule out DNA contamination, establish chromosome copy number, and assess for deletions and duplications. This analysis does not differentiate between euploid and balanced inversion 9 carriers. Accordingly, the embryo results were classified as “unbalanced” if a chromosome abnormality was related to the parental inversion, “euploid” for normal and/or balanced without other aneuploidy, or “sporadic aneuploid” if other chromosomes were abnormal, as previously described [17]. Retrospective review of results was also performed for a control population of embryos from maternal age–matched couples who were referred for 24 chromosome PGT-A and are not known to carry a chromosome rearrangement. In this cohort, PGT-A was also performed using Illumina Cyto12 SNP microarrays with Parental Support^TM^ [16].

The cycle data was deidentified for research purposes and the study was determined to be exempt from Institutional Review Board review (Ethical & Independent Review Services #10806-08).

## Results

Fifty-two TE biopsy samples were received for analysis from seven couples who each underwent a single IVF cycle with PGT-SR for a familial inversion 9 variant with concurrent 24 chromosome PGT-A. Six individuals (five females and one male) were reported to carry an inv(9)(p11q13) and one male was reported to carry an inv(9)(p12q13). Referral indications included parental chromosome rearrangement (*n* = 7), advanced maternal age (*n* = 3), and recurrent pregnancy loss (*n* = 1). The mean maternal age per embryo, calculated by multiplying the maternal age by the number of embryos tested per couple, divided by the total number of embryos tested, was 33.3 years (range 21–39 years). This calculation was performed to ensure that our selected control group had the same proportion of embryos from each maternal age as the inversion 9 study group. All TE samples produced results (see Table 1). None of the tested samples had deletions or duplications related to the parental inversion 9 variant. The overall euploid rate was 61.5 ± 6.7% (32/52) and aneuploid rate was 38.5 ± 6.7% (20/52).

**Table 1:** Embryo results

Case	Parental karyotype	Maternal age (years)	Number of embryos tested	Unbalanced translocation rate (%)	Sporadic aneuploidy rate (%)	Euploid RATE (%)
Case 1	46,XX,inv(9)(p11q13)	35.1	8	0	0	100
Case 2	46,XX,inv(9)(p11q13)	31.1	13	0	30.8	69.2
Case 3	46,XY,inv(9)(p12q13)	21.2	5	0	0	100
Case 4	46,XX,inv(9)(p11q13)	39.2	8	0	50	50
Case 5	46,XY,inv(9)(p11q13)	37.5	3	0	100	0
Case 6	46,XX,inv(9)(p11q13)	34.8	11	0	63.6	36.4
Case 7	46,XX,inv(9)(p11q13)	32.8	4	0	50	50
TOTAL	N/A	33.3 (average per embryo)	52	0	61.5	38.5

A retrospective review of PGT-A results was performed on 2,000 day 5/6 embryo samples from 409 maternal age–matched couples who are not known to carry a chromosome rearrangement (mean maternal age per embryo 33.2 years; range 20–39 years). Patients were referred for PGT-A for reasons including prior failed IVF cycle, recurrent pregnancy loss and advanced maternal age, and excluding parental chromosome rearrangement. Results were obtained on 1944 (97.2%) samples, with a euploid rate of 59.3 ± 1.1% (1153/1944) and aneuploid rate of 40.7 ± 1.1% (791/1944). A comparison of the embryo results from pericentric inversion 9 carriers to those of maternal age–matched controls is shown in Table 2.

**Table 2:** Embryo results of inversion 9 variant carriers compared with maternal age–matched controls*

	Inversion 9 carriers	Controls
Maternal age (years)	33.3 ± 5.9	33.2 ± 4.5
Embryos analyzed (*n*)	7.4 ± 3.7	4.9 ± 3.5
Euploid rate (%)	61.5 ± 6.7%	59.3 ± 1.1%
Aneuploid rate (%)	38.5 ± 6.7%	40.7 ± 1.1%

*Numbers represent mean ± standard deviation

## Discussion

This study reports the PGT-SR SNP microarray analysis results for 52 embryos with the indication of a parental pericentric inversion 9 variant. To our knowledge, this is the first publication to report on the unbalanced inversion rates in day 5/6 embryos from couples with a heterozygous carrier of an inv(9)(p11q13) or inv(9)(p12q13) variant. Importantly, chromosome microarray analysis revealed no inherited unbalanced structural rearrangements in the embryo samples from couples with a pericentric 9 variant. Additionally, we did not observe an increase in aneuploidy rates associated with the inversion 9 variant compared with a group of maternal age–matched patients who pursued 24 chromosome PGT-A and are not known to carry a structural rearrangement.

While pericentric inversions, in general, can be associated with unbalanced chromosome rearrangements, we are unaware of any published reports of inherited deletions or duplications being associated with the pericentric inversion 9 variant. Muthuvel et al. [18] made an unfounded claim that IVF with PGD (now referred to as PGT-SR) is desirable for inversion 9 variants based on the report of one woman with unexplained infertility who was found to carry this inversion. However, PGT-SR was not performed for this patient as she opted to pursue an IVF cycle with an oocyte donor. Young et al. [14] reported on aneuploidy rates via PGT-A in embryos of pericentric inversion 9 carriers and noted that, incidentally, one recombinant embryo with an unbalanced inv(9) was detected. However, the copy number plot for the embryo does not show the expected terminal deletion and duplication distal to the inversion, rather, evidence of an interstitial copy number gain which is not consistent with recombinant forms of the inversion. Thus, the finding is inconsistent with a classic unbalanced form of the inversion, and we presume is unrelated to the inversion 9 carrier status of the parent and more likely a fortuitous finding. Some authors have proposed that pericentric inversions could also interfere with the pairing of homologous chromosomes during meiosis. Amiel et al. [19] described this effect in the sperm of a man with a pericentric inversion 9; however, he also carried constitutive heterochromatin (9qh+), which the authors postulate to be the cause of the increased rate of sperm disomy found in their study. Collodel et al. [7], hypothesized that the inversion 9 variant could have an effect on meiotic segregation; however, while they reported a trend of increased disomy rates in infertile men identified to carry a chromosome 9 inversion, it was not statistically different than what was seen in their fertile controls. Colls et al. [20], also studied 314 sperm from a man who carried the inversion 9 variant and found no increased risk to produce chromosomally abnormal gametes. Furthermore, Young et al. [14] reported on the aneuploidy rates in embryos from 28 patients who carried the inversion 9 variant and demonstrated that a similar rate of aneuploidy was seen in a group of age-matched controls. In our analysis using PGT-SR, none of the embryo samples contained unbalanced forms of the inversion, and we also did not observe an interchromosomal effect associated with the inversion 9 variant, as no increase in aneuploidy rates were observed in the embryos from inversion 9 carriers compared with embryos from maternal age–matched controls.

In the current study, one couple had a referral indication of recurrent pregnancy loss. Prior reports have suggested an association of the inversion 9 variant with infertility and/or miscarriage. Kumar et al. [21] reported the incidence of the inversion 9 variant to be significantly higher in an infertile population compared with a general population, and Daya et al. [9] reported recurrent abortion to be more common in individuals carrying the common 9 inversion; however, no statistical analyses were performed in these studies. Kumar et al. [21] also reported the inversion 9 variant to occur more frequently with male infertility. This finding was also reported by Mozdarani et al. [6], and the authors questioned whether this variant could produce spermatogenic disturbances. However, conflicting results were reported by Sipek et al. [1]. In a large patient cohort, an overall incidence of the inversion 9 variant was found to be greater in females than males, but the finding was not statistically significant (*p* = 0.18). In the study group of individuals with “idiopathic reproductive failure,” the proportion of inversion 9 carriers was reported to be greater in females compared with males, which was statistically different (*p* = 0.0039). Whereas, Dana et al. [22] showed no statistical difference between the rates of the inversion 9 variant in male vs. female carriers with a history of infertility (*p* = 0.343). Nonaka et al. [23] also showed no statistical difference between the rates of the inversion 9 variant in male vs. female carriers with a history of recurrent pregnancy loss (*p* = 0.175). Furthermore, both Dana et al. [22] and Kosyakova et al. [24] reported no evidence for a correlation between the inversion 9 variant and infertility. Similarly, Nonaka et al. [23] studied the pregnancy outcomes in couples with recurrent pregnancy loss where one partner carried an inversion 9 variant and reported that this chromosome finding has no adverse implication on subsequent pregnancies. Karyotyping on villi from products of conception samples from these patients also did not identify any miscarriages with an unbalanced form of the inversion. Our study results are in agreement with these publications, as unbalanced forms of the inversion and increased aneuploidy rates were not identified. Thus, we found no evidence to support that the inversion 9 variant carries a direct association with infertility and/or miscarriage. This supports further medical workup in individuals known to carry this chromosome variant who are evaluated for infertility or pregnancy loss to determine an underlying cause for their reproductive problems.

Patients who are identified to carry a balanced chromosome rearrangement are faced with various reproductive decisions. These choices for the inversion 9 variant are complicated by conflicting literature reporting potential risks associated with this otherwise documented benign population variant. The question of whether the inversion 9 variant could be a predisposing factor for non-disjunction or an interchromosomal effect has been addressed by our analysis which demonstrates no increased risk for unbalanced rearrangements or aneuploidy associated with this chromosome finding. Thus, an inversion 9 variant alone should not be considered an indication for PGT-SR with an IVF cycle. Furthermore, clinicians treating patients with infertility and/or history of miscarriage should pursue additional medical workup to establish causality for reproductive concerns in individuals with the common inversion 9 variant.

In summary, the results of this study indicate that the inversion 9 variant is not associated with an increased risk for unbalanced chromosome products or overall aneuploidy rates. Until larger studies of IVF with PGT-SR for the inversion 9 variant are performed, the findings of the present study can be used to aid in the patient counseling for PGT-SR for common inversion 9 variants. Overall, aneuploidy rates in embryos from couples with a variant inversion 9 carrier are expected to vary based on maternal age and potentially other patient characteristics, as they would for patients who do not carry a balanced chromosome rearrangement.

## References

[1] Šípek A, Panczak A, Mihalová R, Hrčková L, Suttrová E, Sobotka V (2015). Pericentric inversion of human chromosome 9 epidemiology study in Czech males and females. Folia Biol (Praha).

[2] Gardner RJM, Sutherland GR (2004). Chromosome abnormalities and genetic counseling. 3rd ed..

[3] Anton E, Blanco J, Egozcue J, Vidal F (2005). Sperm studies in heterozygote inversion carriers: A review. Cytogenet Genome Res.

[4] Harper PS (2004). Practical genetic counselling. 6th ed..

[5] Verma RS (1988). Heterochromatin: molecular and structural aspects.

[6] Mozdarani H, Meybodi A, Karimi H (2009). Impact of pericentric inversion of Chromosome 9 [inv (9) (p11q12)] on infertility. Indian J Hum Genet.

[7] Collodel G, Moretti E, Capitani S, Piomboni P, Anichini C, Estenoz M (2006). TEM, FISH and molecular studies in infertile men with pericentric inversion of chromosome 9. Andrologia.

[8] Sasagawa I, Ishigooka M, Kubota Y, Tomaru M, Hashimoto T, Nakada T (1998). Pericentric inversion of chromosome 9 in infertile men. Int Urol Nephrol.

[9] Daya S (2006). Issues in the etiology of recurrent spontaneous abortion. Curr Opin Obstet Gynecol.

[10] Stanojević M, Stipoljev F, Koprčina B, Kurjak A (2000). Oculo-auriculo-vertebral (Goldenhar) spectrum associated with pericentric inversion 9: Coincidental finding or etiologic factor?. J Craniofac Genet Dev Biol.

[11] Sotoudeh A, Rostami P, Nakhaeimoghadam M, Mohsenipour R, Rezaei N (2017). Pericentric inversion of chromosome 9 in an infant with ambiguous genitalia. Acta Med Iran.

[12] Gürel SA (2015). Prenatal diagnosis of congenital hallux varus deformity associated with pericentric inversion of chromosome 9. J Obstet Gynaecol Res.

[13] Starke H, Seidel J, Henn W, Reichardt S, Volleth M, Stumm M (2002). Homologous sequences at human chromosome 9 bands p12 and q13-21.1 are involved in different patterns of pericentric rearrangements. Eur J Hum Genet.

[14] Young D, Klepacka D, McGarvey M, Schoolcraft WB, Katz-Jaffe MG (2019). Infertility patients with chromosome inversions are not susceptible to an inter-chromosomal effect. J Assist Reprod Genet.

[15] Teo SH, Tan M, Knight L, Yeo SH, Ng I (1995). Pericentric inversion 9--incidence and clinical significance. Ann Acad Med Singap.

[16] Johnson DS, Gemelos G, Baner J, Ryan A, Cinnioglu C, Banjevic M (2010). Preclinical validation of a microarray Method for full molecular karyotyping of blastomeres in a 24-h protocol. Hum Reprod.

[17] Idowu D, Merrion K, Wemmer N, Mash JG, Pettersen B, Kijacic D (2015). Pregnancy outcomes following 24-chromosome preimplantation genetic diagnosis in couples with balanced reciprocal or Robertsonian translocations. Fertil Steril.

[18] Muthuvel A, Ravindran M, Chander A, Subbian C (2016). Pericentric inversion of chromosome 9 causing infertility and subsequent successful in vitro fertilization. Niger Med J.

[19] Amiel A, Sardos-Albertini F, Fejgin MD, Sharony R, Diukman R, Bartoov B (2001). Interchromosomal effect leading to an increase in aneuploidy in sperm nuclei in a man heterozygous for pericentric inversion (inv 9) and C-heterochromatin. J Hum Genet.

[20] Colls P, Blanco J, Martínez-Pasarell O, Vidal F, Egozcue J, Márquez C (1997). Chromosome segregation in a man heterozygous for a pericentric inversion, inv(9)(p11q13), analyzed by using sperm karyotyping and two-color fluorescence in situ hybridization on sperm nuclei. Hum Genet.

[21] Kumar M, Thatai A, Chapadgaonkar SS (2012). Homozygosity and heterozygosity of the pericentric inversion of chromosome 9 and its clinical impact. J Clin Diagn Res.

[22] Dana M, Stoian V (2012). Association of pericentric inversion of chromosome 9 and infertility in romanian population. Maedica (Buchar).

[23] Nonanka T, Takahashi M, Nonaka C, Enomoto T, Takakuwa K (2019). The analysis of chromosomal abnormalities in patients with recurrent pregnancy loss, focusing on the prognosis of patients with inversion of chromosome (9). Reprod Med Biol.

[24] Kosyakova N, Grigorian A, Liehr T, Manvelyan M, Simonyan I, Mkrtchyan H (2013). Heteromorphic variants of chromosome 9. Mol Cytogenet.

